# Taxonomic determination of the cryptogenic red alga, *Chondria tumulosa* sp. nov., (Rhodomelaceae, Rhodophyta) from Papahānaumokuākea Marine National Monument, Hawai‘i, USA: A new species displaying invasive characteristics

**DOI:** 10.1371/journal.pone.0234358

**Published:** 2020-07-07

**Authors:** Alison R. Sherwood, John M. Huisman, Monica O. Paiano, Taylor M. Williams, Randall K. Kosaki, Celia M. Smith, Louise Giuseffi, Heather L. Spalding

**Affiliations:** 1 School of Life Sciences, University of Hawaiʻi, Honolulu, HI, United States of America; 2 Department of Biodiversity, Western Australian Herbarium, Conservation and Attractions, Kensington, WA, Australia; 3 Department of Biology, College of Charleston, Charleston, SC, United States of America; 4 NOAA, Papahānaumokuākea Marine National Monument, Honolulu, HI, United States of America; 5 NOAA, Pacific Islands Fisheries Science Center, Honolulu, HI, United States of America; CIIMAR Interdisciplinary Centre of Marine and Environmental Research of the University of Porto, PORTUGAL

## Abstract

Survey cruises by the National Oceanic and Atmospheric Administration (NOAA) in 2016 and 2019 yielded specimens of an undetermined red alga that rapidly attained alarming levels of benthic coverage at Pearl and Hermes Atoll, Papahānaumokuākea Marine National Monument, Hawaiʻi. By 2019 the seaweed had covered large expanses on the northeast side of the atoll with mat-like, extensive growth of entangled thalli. Specimens were analyzed using light microscopy and molecular analysis, and were compared to morphological descriptions in the literature for closely related taxa. Light microscopy demonstrated that the specimens likely belonged to the rhodomelacean genus *Chondria*, yet comparisons to taxonomic literature revealed no morphological match. DNA sequence analyses of the mitochondrial COI barcode marker, the plastidial *rbc*L gene, and the nuclear SSU gene confirmed its genus-level placement and demonstrated that this alga was unique compared to all other available sequences. Based on these data, this cryptogenic seaweed is here proposed as a new species: *Chondria tumulosa* A.R.Sherwood & J.M.Huisman sp. nov. *Chondria tumulosa* is distinct from all other species of *Chondria* based on its large, robust thalli, a mat-forming tendency, large axial diameter in mature branches (which decreases in diameter with subsequent orders of branching), terete axes, and bluntly rounded apices. Although *C*. *tumulosa* does not meet the criteria for the definition of an invasive species given that it has not been confirmed as introduced to Pearl and Hermes Atoll, this seaweed is not closely related to any known Hawaiian native species and is of particular concern given its sudden appearance and rapid increase in abundance in the Papahānaumokuākea Marine National Monument; an uninhabited, remote, and pristine island chain to the northwest of the Main Hawaiian Islands.

## Introduction

Invasive seaweeds are well known in the Main Hawaiian Islands, with the most problematic of these being the red algae *Acanthophora spicifera* (M.Vahl) Børgesen, *Gracilaria salicornia* (C.Agardh) E.Y.Dawson, *Hypnea musciformis* (Wulfen) J.V.Lamouroux, members of the *Kappaphycus/Eucheuma* complex, and the green alga *Avrainvillea lacerata* J.Agardh (previously recorded as *A*. *amadelpha* (Montagne) A.Gepp & E.S.Gepp) [1,2]. The origins of these seaweeds in the Hawaiian Islands vary, with *A*. *spicifera* reportedly arriving on a fouled hull of a naval fuel barge that arrived in Pearl Harbor Naval Station in 1950 [3] and the remainder of the red algae escaping from aquaculture trials of imported material [1]. The origins of the Hawaiian populations of the green alga, *A*. *lacerata*, are unknown; some possibilities include that the alga is an indigenous member to the flora or an early introduction before collections of even shallow water algae were first made in about 1826 [4]. *A*. *lacerata* has now spread into shallow waters in recent decades from deeper habitats [2,5,6,7]. More recently, *Avrainvillea erecta* (Berkeley) A.Gepp & E.S.Gepp has been recorded on O‘ahu and Maui as an introduced species, and continues to rapidly increase in abundance and distribution [6]. In contrast to the Main Hawaiian Islands, reports of nuisance algae in Papahānaumokuākea Marine National Monument (PMNM) (the Northwestern Hawaiian Islands) are far fewer, and no invasive behavior by non-indigenous seaweeds has been observed. In 2008, a non-persistent bloom of the green alga *Boodlea composita* (Harvey) Brand was reported at Kure and Midway Atolls [8], and extensive collections of an unidentified species of the red alga *Hypnea* have been examined from specimens derived from lobster traps from Maro Reef and Mokumanamana (Necker Island) [9]; however, these have not been characterized or confirmed as invasive species.

A widespread mat-forming alga was first observed in 2016 by National Oceanic and Atmospheric Administration (NOAA) researchers who were conducting routine monitoring surveys of coral reefs at Pearl and Hermes Atoll (PHA), PMNM. It was identified by eye as a red alga, and was noted to form mat-like growths that extended for several square meters, with detached pieces exhibiting “tumbleweed”-like morphologies. Surveys conducted in July/August and September 2019 confirmed the presence and spread of the alga, and documented it from 1–19 m depths around the northern, western, and eastern sides of PHA. Here we employ morphological and molecular characterization of samples, in combination with taxonomic comparisons in the literature, to provide a taxonomic identification for this rapidly spreading, cryptogenic nuisance alga.

## Materials and methods

Specimens of *Chondria* were collected in September 2016, July/August 2019, and September 2019 from PHA, PMNM, Hawai‘i, USA, as part of surveys conducted by NOAA divers (S1 Table). Field work was conducted under PMNM permits PMNM-2015-029 and PMNM-2018-029 to R. Kosaki. Samples were collected by SCUBA for molecular characterization and were cleaned of epiphytes and placed in silica gel desiccant. Samples were preserved for morphological characterization by fixing in 4% formalin/seawater. Morphological investigations were conducted by hand sectioning with a double-edged razor blade, staining with 0.5% aniline blue or 0.05% ruthenium red, and mounting in 30–50% Karo™. Photomicrographs were taken on a Zeiss AxioImager A1 compound light microscope (Pleasanton, CA) with an Infinity2-1RC digital camera (Lumenera Corporation, Ottawa, Ontario, Canada). Morphological characters that were measured or determined as described above were used to compare the new specimens to all previously described taxa within the genus *Chondria* to determine if they represented a currently recognized taxon, or an undescribed species.

Specimens were extracted for genomic DNA using an OMEGA E.Z.N.A.® Plant DNA DS Kit (OMEGA Biotek, Norcross, GA, USA). A portion of the COI DNA barcode marker (cytochrome oxidase subunit I, 658 bp) was amplified using the GazF1 and GazR1 primers [8] or the GazF2 and Gaz R2 primers [10,11]. The *rbc*L (ribulose-1,5-bisphosphate carboxylase/oxygenase large subunit, 1,442 bp) marker was amplified as two overlapping fragments using the primer pairs rbcLF7 and rbcLJNR1 [12,13] and rbcLF762 and rbcLR1442 [14]. The SSU (small subunit ribosomal DNA, 1,038 bp) amplification was amplified in two overlapping fragments with primer pairs SR1 and SR5, SR4 and SR9 [15]. Successful PCR products were submitted for sequencing to the GENEWIZ Corporation (South Plainfield, New Jersey, USA). Raw sequence reads for each gene were assembled, edited, and aligned using the MUSCLE v. 3.8.425 plug-in [16] in Geneious Prime 2019.1.3 (http://www.geneious.com) with related sequences from GenBank and the Barcode of Life Datasystems (BOLD) Database (S2 Table). BLAST comparisons to GenBank and BOLD were employed to examine taxonomic associations of the individual sequences, and to confirm that all analyzed sequences were from red algae. DNA barcode analysis of the COI sequences was performed by constructing a neighbor-joining (NJ) framework based on Kimura-2-parameter distances using MEGA X [17]. Reference *rbc*L and SSU sequences against which to compare the newly generated sequence data were selected by searching for and including all *Chondria* and *Neochondria* sequences on GenBank, as well as representation of the remaining 20 tribes of the Rhodomelaceae, to the extent possible. This yielded comparative data for 18 of the 20 remaining tribes for the *rbc*L marker, and 19 for the SSU marker. Outgroups were selected following Díaz-Tapia et al. [18]. For the *rbc*L and SSU phylogenetic analyses, sequences were aligned with reference sequences downloaded from GenBank and analyzed with PartitionFinder v. 1.1.1 [19]. Six COI sequences from the Hawaiian Rhodophyta Biodiversity Survey [20] were also included in the analyses to determine whether the new collections matched any previously known members of the genus. New *rbc*L sequences were also generated for as many of these previously collected Hawaiian specimens as possible, and included in the phylogenetic analyses. Maximum Likelihood (ML) analyses were performed on all alignments using RAxML-HPC2 on XSEDE v. 8.2.10 [21] via the CIPRES gateway [22] with 1,000 bootstrap replicates, and using the GTRCAT model. Bayesian inference was performed using the MrBayes plug-in v. 3.2.6 [23] through Geneious Prime 2019.1.3 (http://www.geneious.com) using four chains of Metropolis-coupled Markov Chain Monte Carlo for 1,000,000 generations and sampling every 100 generations; 100,000 chains were removed as burn-in prior to determining posterior probabilities.

### Nomenclature

The electronic version of this article in Portable Document Format (PDF) in a work with an ISSN or ISBN will represent a published work according to the International Code of Nomenclature for algae, fungi, and plants, and hence the new names contained in the electronic publication of a PLOS article are effectively published under that Code from the electronic edition alone, so there is no longer any need to provide printed copies.

In addition, new names contained in this work have been submitted to IPNI, from where they will be made available to the Global Names Index. The IPNI LSIDs can be resolved and the associated information viewed through any standard web browser by appending the LSID contained in this publication to the prefix http://ipni.org/. The online version of this work is archived and available from the following digital repositories: PubMed Central, LOCKSS.

## Results

Preliminary microscopical analysis confirmed the PHA alga as a member of the red algal genus *Chondria*, in the family Rhodomelaceae (Ceramiales). This identification was based on cross sections of the alga revealing a central axial cell surrounded by five pericentral cells, which were evident well beyond the apices of the thallus, the pericentral cells being surrounded by a compact, cellular cortex to form a branched, terete thallus, and the presence of tetrahedral tetrasporangia near the apices of secondary branches. Using this initial genus-level identification, DNA sequence frameworks (COI) and phylogenies (*rbc*L and SSU) were constructed with related representation for the three markers. All newly generated sequence data have been submitted to GenBank (COI: MT039621—MT039626; *rbc*L: MT039601—MT039620; SSU: MT039627—MT039630).

### DNA barcoding and molecular phylogenetic analyses

**Fig 1:**
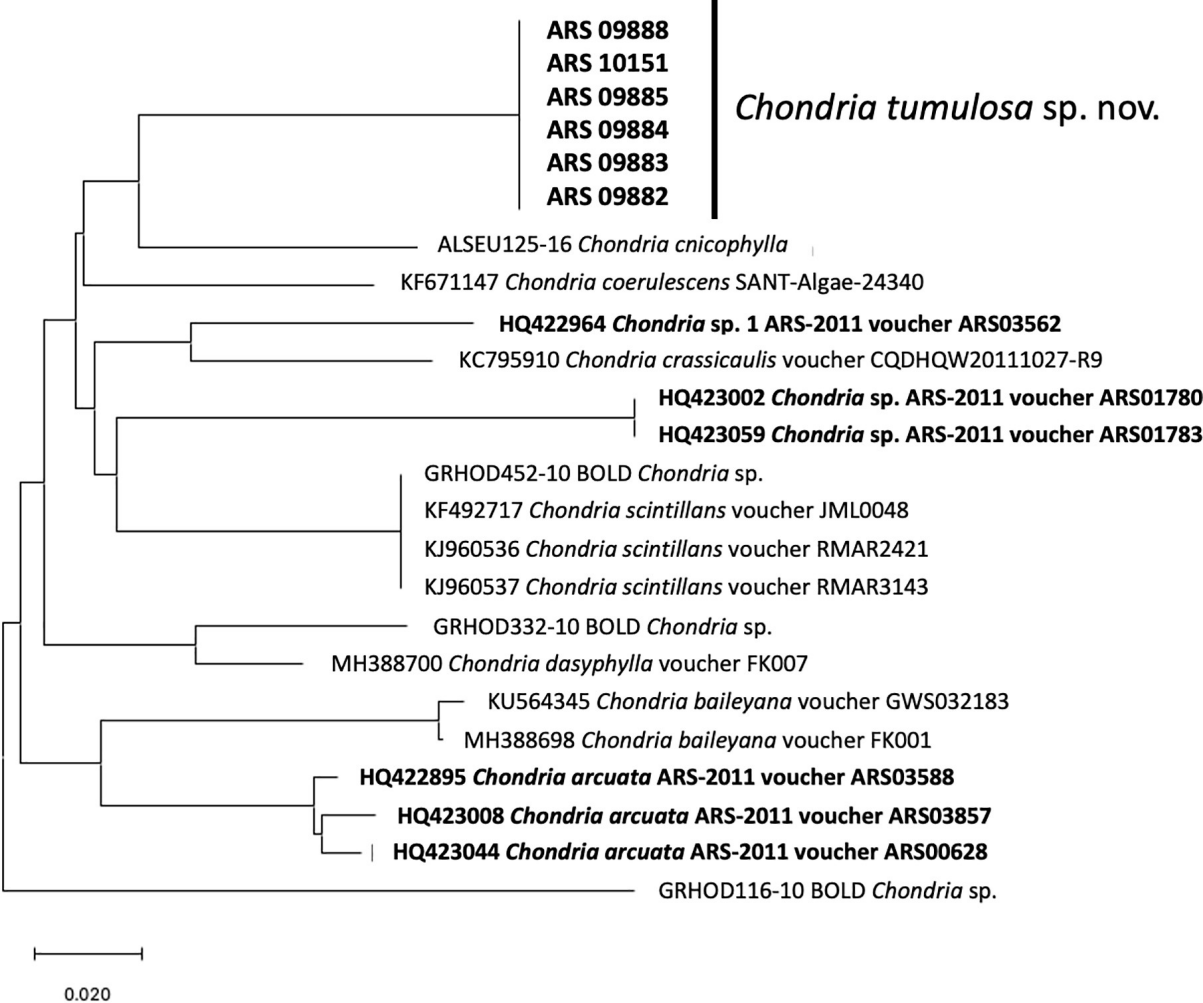
Neighbor-joining phylogram (K2P distances) of COI sequences of *Chondria*. The phylogram analysis demonstrates the sequence divergence of *C*. *tumulosa* sp. nov. samples from other represented members of the genus, and illustrates that the newly analyzed specimens from Pearl and Hermes Atoll do not match previously characterized specimens in the genus. Additional Hawaiian specimens are shown in bold. Scale bar = substitutions per site.

Six samples of the PHA alga were sequenced for the COI barcoding region: One collected in 2016, and five collected in 2019. All six samples were identical in sequence. Using the BLAST algorithm on GenBank and BOLD, this sequence was identified as likely belonging to the genus *Chondria*, although a species-level match was not found. The NJ analysis demonstrated that the *Chondria* sequence was unique from all other available sequences for the genus, including those for other Hawaiian species of *Chondria* (Fig 1).

**Fig 2:**
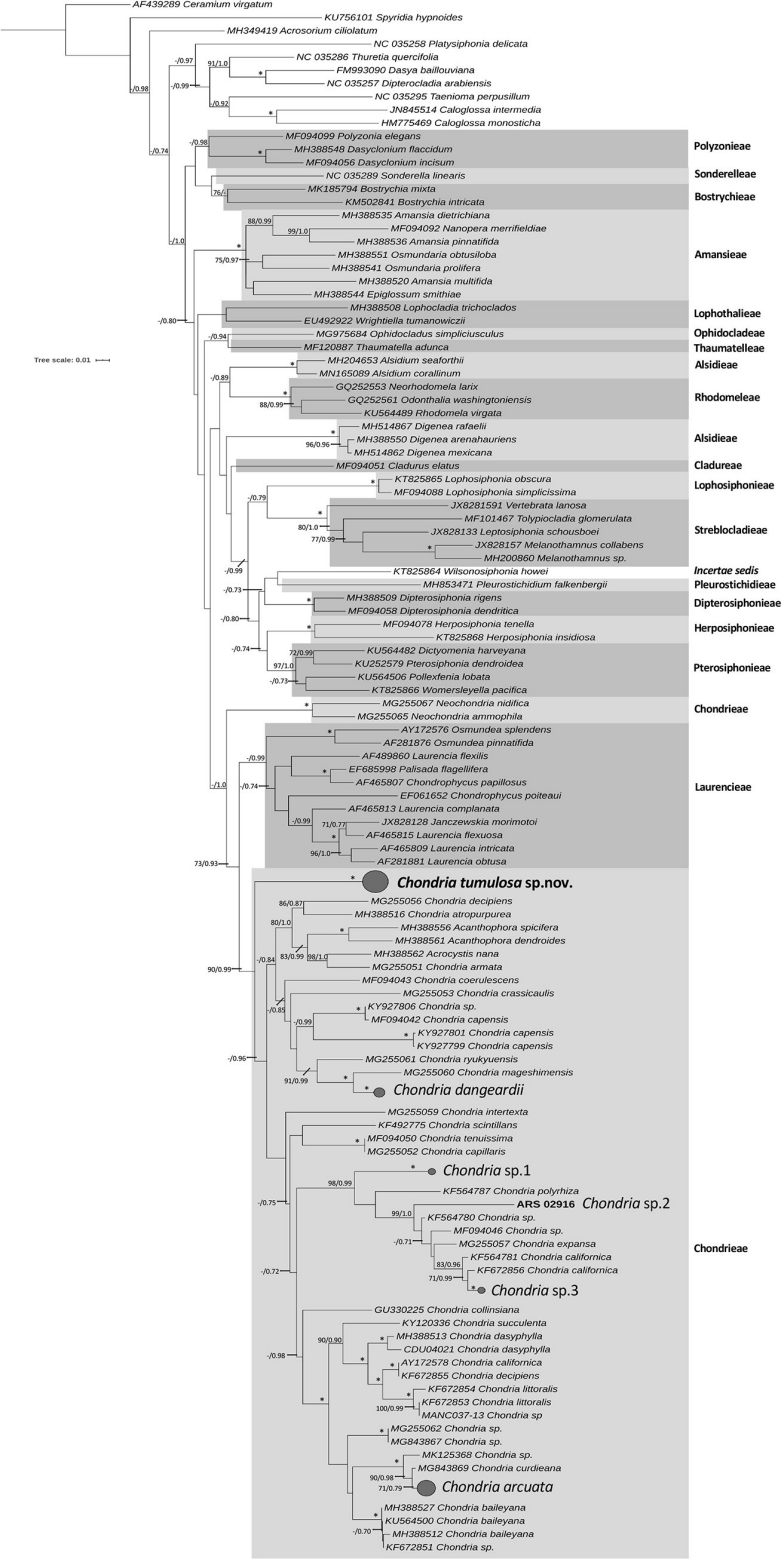
Maximum likelihood analysis of the red algal genus *Chondria* based on *rbc*L. *Chondria tumulosa* sp. nov. is resolved as a distinct lineage within the tribe Chondrieae based on available *rbc*L sequences for representatives of the Rhodomelaceae, and is shown to not match previously characterized Hawaiian samples. Numbers along branches indicate nodal support (first value = bootstrap support, second value = Bayesian posterior probabilities). Nodes with full support are indicated with an asterisk. Hawaiian specimens are shown in bold. Scale bar = substitutions per site.

The molecular phylogenetic analysis of the *rbc*L marker for representatives of 18 of the 21 tribes of the Rhodomelaceae, is presented in Fig 2. Seven samples of the PHA alga were sequenced for *rbc*L (all from 2019) and were identical in sequence, and analysis of this sequence resolved it as sister to the other available sequences for the genus *Chondria* (Fig 2). Additionally, sequences of *Acanthophora* and *Acrocystis* were resolved within the *Chondria* clade. Five additional species-level clades of Hawaiian *Chondria* were resolved in the analysis from samples sequenced for the *rbc*L marker from the 2010 Hawaiian Rhodophyta Biodiversity Survey. Two of the five Hawaiian *Chondria* lineages corresponded morphologically to what are referred to as *C*. *arcuata* Hollenberg and *C*. *dangeardii* E.Y.Dawson, while vouchers were inconclusive for the taxonomic assignment of the remaining three lineages (sp. 1–3). All five Hawaiian *Chondria* clades were clearly distinct from the PHA species (Fig 2).

**Fig 3:**
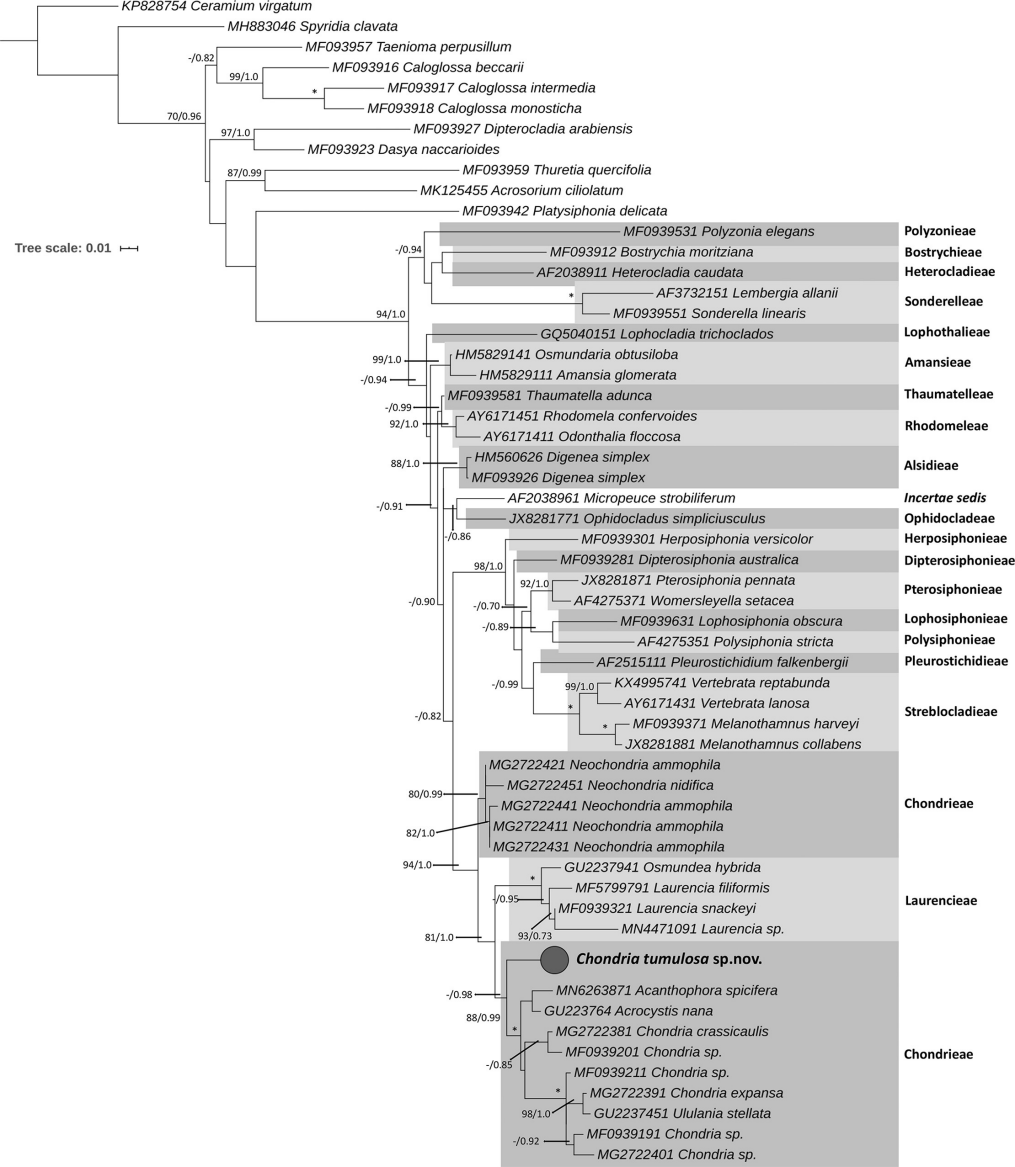
Maximum likelihood analysis of the red algal genus *Chondria* based on nuclear SSU. *Chondria tumulosa* sp. nov. is resolved as a distinct clade within the tribe Chondrieae, based on sequences available SSU sequences for representatives of the Rhodomelaceae. Numbers along branches indicate nodal support (first value = bootstrap support, second value = Bayesian posterior probabilities). Nodes with full support are indicated with an asterisk. Hawaiian specimens are shown in bold. Scale bar = substitutions per site.

Although fewer reference sequences were available for the SSU marker for the genus *Chondria* overall, representation of the tribes of the Rhodomelaceae by SSU was slightly better than for *rbc*L, and included 19 of 21 currently recognized tribes. Phylogenetic analysis of SSU sequences demonstrated that the PHA *Chondria* was resolved as a distinct lineage that was sister to other sequences of *Chondria*, although support values for the relationship of this clade to others within the genus were not high enough to allow confidence in the phylogenetic positioning of the samples (Fig 3). As for the *rbc*L analysis, sequences of other genera of the Chondrieae (e.g., *Acanthophora*, *Acrocystis*, *Ululania*) resolved within *Chondria*.

### Taxonomic analyses

Of the 175 infrageneric taxonomic names currently listed on AlgaeBase, 60 are now considered to be members of genera other than *Chondria*, and are thus discounted [24]. A further 23 taxa are currently considered to be synonymous with other species in the genus *Chondria* [24]. The original descriptions (or, in a few cases, subsequent monographs that included detailed taxonomic analysis) were obtained and compared for all remaining described and currently recognized species within the genus *Chondria*. A satisfactory match of taxonomic characters for the PHA *Chondria* species and these 92 currently recognized species and infraspecific taxa of *Chondria* was not found. Based on the unique combination of morphological features for the PHA alga, in combination with the DNA barcoding and molecular phylogenetic results, we propose this taxon as a new species within the genus *Chondria*.

### ***Chondria tumulosa* A.R.Sherwood et J.M.Huisman sp. nov.** (Fig 4A–4K)

[urn:lsid:ipni.org:names:XXXX]

**Fig 4:**
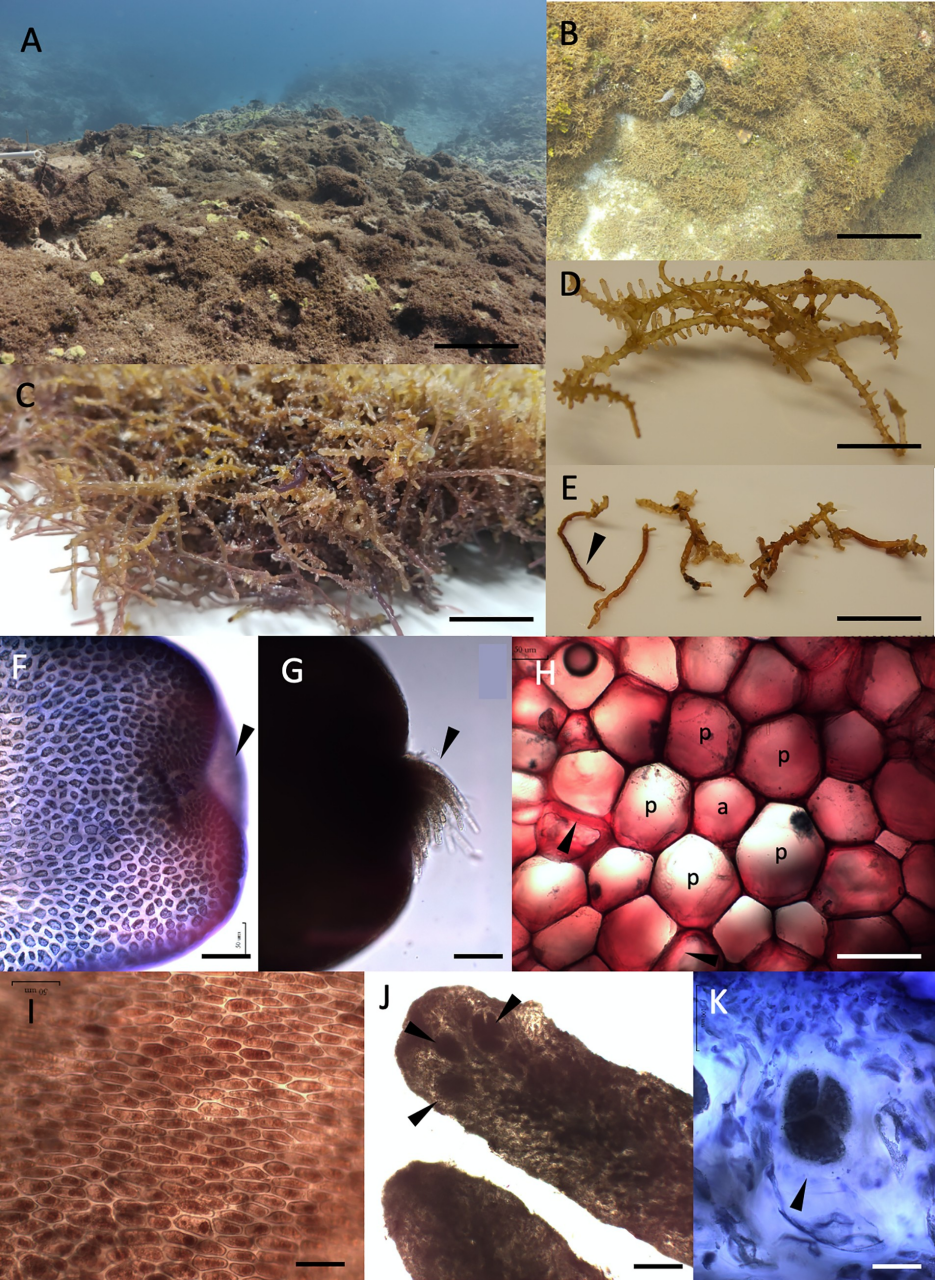
Morphological features of *Chondria tumulosa* sp.nov. A. View of *Chondria* overgrowing the coral reef at Pearl and Hermes Atoll (PHA) at 12 m depth, Papahānaumokuākea Marine National Monument, Hawai‘i, USA. Scale bar = 30 cm. B. Close view of *Chondria tumulosa* at PHA. Scale bar = 10 cm. C. Macroscopic view of the edge of a mat of *Chondria tumulosa*, illustrating the intertwined and mat-like growth of the alga, and the color variations from yellowish to purple. Scale bar = 2 cm. D. Close up view of several main axes that were separated from the upper surface of a mat, illustrating the numerous secondary and tertiary branches from the main axis. Scale bar = 5 mm. E. Close up view of several main axes that were separated from the lower surface of a mat, illustrating the darker color and slender haptera (arrow) used for attachment of the mat to the substratum. Scale bar = 5 mm. F. A branch apex, focused internally to illustrate the apical depression, or pit. Scale bar = 50 μm. G. A branch tip, focused to illustrate trichoblasts emerging from the pit. Scale bar = 50 μm. H. Cross section of *Chondria tumulosa* stained with ruthenium red, demonstrating the typical rhodomelacean structure, with a central axial cell (a) surrounded by five pericentral cells (p). Cell wall thickenings are indicated in some cells (arrows) outside of this central structure. Scale bar = 50 μm. I. Surface view of cortical cells in the epidermal layer. Scale bar = 50 μm. J. Tetrasporangia developing towards the apices of secondary and tertiary axes. Scale bar = 200 μm. K. Close-up of a tetrahedrally divided tetrasporangium, stained with aniline blue. Scale bar = 100 μm.

#### Diagnosis

Differing from all other species of *Chondria* based on its large, robust thalli, its mat-forming tendency, large axial diameter (which decreases in diameter with subsequent orders of branching), terete axes, and bluntly rounded apices.

#### Description

Plants forming mounds/mats to many meters in horizontal extent, typically growing on coral, composed of irregularly branched thalli with imbricating axes and numerous anastomoses between branches, loosely attached to the substratum by haptera. Individual thalli 2–7 cm in length, golden-brown to honey-colored, dark brown or purplish maroon, branching to 3–4 orders, the degree of primary and secondary branching varying considerably, often with short lateral branches, these seemingly determinate; axes terete; main axes 1190–1430 μm in diameter, decreasing to (640) 830–1030 μm in lateral branches, and 280–325 μm in next order branches. Young branchlets somewhat constricted at the base. Apices bluntly rounded, with a small pit and occasional emergent trichoblasts. Mature epidermal cells elongate-polygonal in shape, 30–45 μm x 7–13 μm (length:breadth 3.5–4.3) in surface view. Internal rhizoids lacking. Lenticular thickenings occasionally present in medullary cells, often absent. Tetrasporangia immersed at the apices of secondary or tertiary branches, several per apex, to 120–125 μm in diameter x 150–185 μm long, subspherical, tetrahedrally divided. Gametangial reproduction not observed.

#### Holotype

BISH 776132 (ARS 10154, Pearl and Hermes Atoll, Hawai‘i (27º47.3952'N, 175º59.889'W), 12 m depth, 01.VIII.2019, leg. T. Williams (NWHI-879), sheet 1), tetrasporangial plants.

#### Isotypes

BISH 776133 (ARS 10154, Pearl and Hermes Atoll, Hawai‘i (27º47.3952'N, 175º59.889'W), 12 m depth, 01.VIII.2019, leg. T. Williams (NWHI-879), sheet 2, tetrasporangial plants. HAW-43414 (ARS 10154, Pearl and Hermes Atoll, Hawai‘i (27º47.3952'N, 175º59.889'W), 12 m depth, 01.VIII.2019, leg. T. Williams (NWHI-879), sheet 3), tetrasporangial plants.

#### Etymology

Named for the mound-like morphology of the alga at the type locality; adjectival form of the Latin “tumulans”, or a mound.

#### Distribution

Pearl and Hermes Atoll, Papahānaumokuākea Marine National Monument, Hawai‘i, USA, from 1–19 m depth.

#### Specimens examined

ARS 09889, ARS 10151, ARS 10154 (BISH 776130–776133)

#### DNA sequence data

GenBank accessions MT039601-MT039607 (COI), MT039621-MT039626 (*rbc*L), MT039627-MT039630 (SSU).

#### Habit and morphology

The mat- and mound-forming habit of *C*. *tumulosa* was noted at low occurrence in the September 2016 collections and widely observed in the August/September 2019 surveys of PHA, with mats up to 1 m in extent easily removed from the substratum in a single piece (Fig 4A–4C). Mats overgrow and cause mortality to native corals and macroalgae by smothering saxicolous benthic communities. Cascades of the mat overgrow the tops and ridges of spur and groove reefs, creating a homogenous reef landscape. Individual axes were difficult to disentangle from the larger mats, and were composed of primary axes with secondary and tertiary branches that decreased in diameter with each order of branching (Fig 4D). Mats in shallow (~1–3 m) water subject to wave motion were loosely attached to the substratum via multicellular haptera, which were observable as thinner, darker, unbranched, multicellular sections of the thallus on the underside of mats (Fig 4E). Mats in deeper (>3 m) water with less wave motion were strongly attached to the substrate by haptera, with mats more difficult to remove as large pieces. Thalli ranged in color from golden-brownish to honey-colored on the upper sides of mats exposed to higher levels of light, and dark brown or purplish maroon on the undersides of mats, which received lower light levels (Fig 4D, 4E). Axes were terete throughout the thalli, and no compressed regions were observed (Fig 4D and 4E). Apices of branches were bluntly rounded, not tapered (Fig 4F), and in some cases, exhibited tufts of trichoblasts emerging from a pit (Fig 4F and 4G). Cross sections through various points along the apices revealed a typical rhodomelacean arrangement of five pericentral cells surrounding a central axial cell, and occasional cell thickenings (Fig 4H). Cortical cells varied in shape, but in mature portions of the plants were elongate-polygonal in surface view (Fig 4I). Tetrasporangia were abundant in collections from the three cruises (Fig 4J and 4K), however no gametangia or cystocarps were observed.

### Ecology

*Chondria tumulosa* was found on spur and groove reefs, aggregate patch reefs, and hard substrate from the edge of the backreef to forereef, but was not observed within the lagoon interior or at mesophotic depths. The alga’s highest abundance occurred at 10–15 m depth on the forereef, where it formed thick mats up to 18 cm thick that mounded over the reef, smothering coral, native macroalgae, and other organisms. This seaweed covered large areas of reef and was observed in dense patches covering up to several thousand square meters each. Large thick mats were observed abrading and sloughing off the reef, revealing completely bare substrate and dead corals underneath. Coral genera observed being smothered by this alga included native species of *Porites*, *Pocillopora*, *Leptastrea*, *Montipora*, *Cyphastrea*, *Pavona*, *Fungia*, and *Psammocora*. Native fishes were not observed to graze on *C*. *tumulosa* over the period of these dives at PHA.

## Discussion

Five species of *Chondria* were recorded in the most recent floristic treatment of Hawaiian red algae: *C*. *arcuata*, *C*. *dangeardii*, *C*. *minutula* Weber-van Bosse, *C*. *polyrhiza*, and *C*. *simpliciuscula* Weber-van Bosse [25]. Abbott [25] noted, however, that several additional names were recorded in the literature for the Hawaiian flora: *C*. *baileyana* (Montagne) Harvey, *C*. *tenuissima* (Withering) C.Agardh (and some of its varieties) and *C*. *repens* Børgesen (which she updated to *C*. *polyrhiza* Collins et Hervey). Abbott also remarked that only approximately half of the *Chondria* specimens in Hawaiian collections could be identified to species given the lack of reproductive material in the collections. Of the species recorded above, only *C*. *simpliciuscula* bears any morphological similarity to the *Chondria* species collected from Pearl and Hermes Atoll; however, *C*. *simpliciuscula* can be excluded from consideration based on our specimens having larger diameter axes (to 1430 μm versus 900 μm), larger tetrasporangia (to 125 x 185 μm versus to 55 μm diameter) and smaller epidermal cells (30–45 μm x 7–13 μm versus 78–92 x 8–20 μm) [25,26].

Broader comparisons of the PHA *Chondria* yielded no clear match to other described species in the genus. The large, robust, mat-forming tendency of *C*. *tumulosa*, combined with its terete axes and bluntly rounded apices, set it apart from all other currently recognized species. Many *Chondria* species are either small, slender, epiphytic, and/or have flattened axes or pointed apices, all of which are characters that differ markedly from the alga collected from PHA [27,28,29]. However, a few species bear some similarity to the new species and are worthy of more direct comparison. *Chondria transversalis* Børgesen shares a clumped habit and similar apex morphology with *C*. *tumulosa*, but this species is much smaller overall than *C*. *tumulosa*; additionally, the axes of *C*. *transversalis* are all of similar diameter, which contrasts with the sharp decrease in axis diameter with each order of branching in *C*. *tumulosa* [29,30]. *Chondria infestans* (A.H.S.Lucas) A.J.K.Millar also attaches via multicellular haptera; however, it is prostrate with flattened axes, and so quite different in morphology from *C*. *tumulosa* [31,32]. *Chondria littoralis* Harvey has fusiform laterals that taper to base and apex, which differs from the terete and mostly unconstricted axes of our species, although it has been reported as dense and spreading at Key West, Florida, suggesting some similarity in its ability to grow to nuisance or near-nuisance levels [33]. *Chondria hapteroclada* C.K.Tseng is reasonably close in morphology to the PHA alga, but *C*. *hapteroclada* is smaller overall, with axes up to 0.72 mm, and has tetrasporangia that are also reported up to only approximately 90 μm [34], which is substantially smaller than *C*. *tumulosa*. *Chondria tumulosa* shares an irregularly branched habit and large stature with *C*. *telmoensis* E.Y.Dawson, as well as obtuse apices and similar apical pits with emergent trichoblasts. However, *C*. *telmoensis* differs in being loosely and openly branched in habit, in contrast to the clumped, matted habit of *C*. *tumulosa*, in addition to having larger tetrasporangia than *C*. *tumulosa* [35]. Finally, *C*. *densa* P. Dangeard is similar to *C*. *tumulosa* in its abundant and dense growth, and highly branched habit with a tendency to form stolons, but differs in several characters; the axis diameter and tetrasporangia are much larger for *C*. *tumulosa*, while the epidermal cells are smaller [36].

Molecular phylogenetic analysis of *rbc*L gene sequences revealed a tree topology largely consistent with those reported by Sutti et al. [37] and Díaz-Tapia et al. [18]. *Neochondria* was resolved as sister to the Laurencieae and Chondrieae in both this study and Sutti et al. [37] (and was not addressed in Díaz-Tapia et al. [18]), suggesting either that *Neochondria* requires re-assignment to its own tribe, or that the two tribes should be merged. Additionally, as in previous studies, the Laurencieae and Chondrieae (minus *Neochondria*) are resolved as distinct clades [18,37,38]. Within the Chondrieae, *C*. *tumulosa* was resolved as sister to a clade containing all other *Chondria* sequences, indicating that this species does not likely represent a recent evolutionary event within the genus. Nevertheless, morphological analysis of the voucher specimens indicates that these specimens fit within the circumscription of *Chondria*, rather than a different genus. The SSU phylogeny, although consisting of far fewer sequences than the *rbc*L phylogeny, also resolved the Laurencieae and Chondrieae as distinct tribes. As for the *rbc*L analysis, *C*. *tumulosa* was shown to be a member of the genus *Chondria*, and distinct from all other sequenced species within the genus, while recognizing that the proportion of species within *Chondria* that have representative sequence data is still very small (26% for *rbc*L, and only 2% for SSU).

The lack of a close morphological match, combined with the unique molecular signature of the new specimens, and the absence of previous records in the Hawaiian Islands of a similar taxon, indicate that the best path forward is through description of the PHA alga as a new species. Although it remains a possibility that future comparisons of DNA sequence data from other *Chondria* specimens (including type specimens) may reveal identity with *C*. *tumulosa*, this would be easily remedied through taxonomic synonymy. The opposite approach, i.e., force-fitting this nuisance species into a current taxonomic entity, or leaving its species-level affinity unresolved (i.e., as *Chondria* sp.), is not a desirable path, considering the sudden and large ecological impact of this species. Moreover, the taxonomic uncertainty of many of the earlier described species in the genus (a challenge for the entire discipline of phycological systematics) means that, pragmatically, molecular comparisons of all species will never be achieved [39]. By assigning a scientific name and recognizing this taxon as distinct, it can be clearly and unambiguously referenced in the literature assessing its environmental impact, its geographical extent through traditional survey and eDNA detection methods, and attempts to control its spread. This approach is particularly important for species that are studied from remote and not easily accessible locations, such as the PMNM, because opportunities to return, collect, and analyze taxa from this region are very limited [40].

Biological invasions by introduced species have a profound effect on species diversity [41,42,43] and fundamentally shift the ecology of a region by modifying ecosystem processes, community composition, and food-web dynamics [44,45]. However, the exact meaning of the word “invasive” can be unclear with respect to macroalgae [46], particularly when the origin of the alga is undetermined (i.e., “cryptogenic”) and the alga is clearly having a harmful effect on the environment. Executive Order (EO) 13112, discussed by Beck et al. [47], defines an “invasive” species as “an alien species whose introduction does or is likely to cause economic or environmental harm or harm to human health.” In the Executive Summary of the National Invasive Species Management Plan (NISMP) the term “invasive species” is further clarified and defined as “a species that is non-native to the ecosystem under consideration and whose introduction causes or is likely to cause economic or environmental harm or harm to human health.” Williams and Smith [48] define “introduced” as a species introduced beyond its native range by human activities and successfully established; the “invasibility” of a system is the susceptibility of a native community to the establishment of an introduced species. The term “invasive” thus refers to a condition whereby an introduced species becomes excessively abundant, usually causing ecological or economic harm [48,49], where the introduced species acts as a new keystone species, either having a strong impact on the native keystone species or replacing it [49]. The *Chondria* species described herein was observed overgrowing native reef organisms, and replacing native keystone species, which would best be described as a type of invasive behavior. Additional molecular analyses from *Chondria* species throughout the Pacific are needed to confirm whether this is an introduced species to PHA, or a new species that recently and quickly became highly abundant. While others have considered that native algae able to exhibit emerging invasive behaviors, those examples are typically in habitats under extreme stress such as persistent blooms of *Dictyosphaeria cavernosa* (Forsskål) Børgesen on reefs of Kāne‘ohe Bay during extreme periods of land-based sources of pollution [50,51]. Until the origin of *Chondria tumulosa* is clarified, we qualify this species as exhibiting invasive characteristics, given its sudden appearance, rapid overgrowth and ecological harm to the coral reef ecosystem. Potential vectors of introduction for this alga include marine debris and fishing gear as well as hull fouling or ballast water [46,48]. Given the protected nature of the PMNM it will be difficult-to-impossible to confirm the mode of introduction, especially while the biogeographic origin of the seaweed remains a mystery. Nonetheless, the possibility exists that changing ocean and benthic community conditions associated with climate change delivered and enabled this new seaweed to establish and then thrive on PHA reefs. Although studies documenting changes in invasive seaweed distributions with climate change are limited, there is increasing evidence that climate change-related range shifts in seaweed distributions are likely to occur [52], and that benthic habitat transformations related to climate change increase the abundance of invasive seaweeds [53].

Globally, several other species of *Chondria* have been reported as introduced and of concern. For example, *C*. *coerulescens* (J.Agardh) Sauvageau, *C*. *curvilineata* Collins et Hervey, and *C*. *pygmaea* Garbary et Vandermeulen were likely introduced in the Mediterranean in the last several decades [54]. Additionally, the rhodomelacean alga, *Acanthophora spicifera*, is well known and documented as an invasive species in the Main Hawaiian Islands [1], and bears limited morphological similarity to *C*. *tumulans*. However, none of these species has exhibited the rapid abundance, mat formation, and overgrowth observed in *C*. *tumulosa* at PHA. Although *C*. *tumulosa* has only very recently been detected at PHA (2016 and 2019), considerable alarm has been raised over its potential ecological impact (https://www.papahanaumokuakea.gov/new-news/2019/08/15/coral-alga-research/). This species is, to the best of our knowledge, the most persistent of the relatively few nuisance algae reported from PMNM, and its invasive characteristics are deeply concerning for coral reef ecosystem health beyond PHA to the broader region of PMNM. If this algal is a harbinger of changes coming, rapid responses are needed to this new threat [47,48]. Effective responses rely, in part, on accurate taxonomic identification, which we aim to provide through its recognition as *Chondria tumulosa* sp. nov.

## Conclusions

A newly discovered species of macroalga exhibiting invasive characteristics at Pearl and Hermes Atoll, PMNM, Hawai‘i, USA, is described as a new species, *Chondria tumulosa* sp. nov. This new species description is supported by a combination of morphological comparisons to previously described species in the genus, and molecular phylogenetic comparisons to closely related taxa. Observed at PHA since 2016, the rapid increase in areal coverage of the alga in the three years between surveys, combined with its mat-forming abilities and “tumbleweed” fragmentation, led to the realization that this seaweed has the potential to significantly alter the pristine reef ecological structure at this remote atoll and throughout the Hawaiian archipelago if or when it spreads to other islands and atolls. This formal description represents the first step toward understanding the biogeographical origin and ecology of the alga leading to the development of appropriate management recommendations.

## Supporting information

S1 Table Specimens of *Chondria tumulosa* sp. nov. characterized as part of the current study. (DOCX)

S2 Table Accession data for sequences used in phylogenetic analyses. (DOCX)
