# Supplementary material for: Taxonomic determination of the cryptogenic red alga, Chondria tumulosa sp. nov., (Rhodomelaceae, Rhodophyta) from Papahānaumokuākea Marine National Monument, Hawai‘i, USA: A new species displaying invasive characteristics
Source: PLoS One. 2020 Jul 7;15(7):e0234358. doi: 10.1371/journal.pone.0234358 (PMC7340295; doi:10.1371/journal.pone.0234358)
Supplement: S1 Table — (DOCX) [file pone.0234358.s001.docx]

**S1 Table. Specimens of *Chondria tumulosa* sp. nov. characterized as part of the current study.**

| **Sherwood Lab collection** | **Herbarium Accession(s)** | **Collection information (latitude/longitude in decimal degrees)** | **GenBank accession (COI)** | **GenBank accession (*rbcL*)** | **GenBank accession (SSU)** |
| --- | --- | --- | --- | --- | --- |
| ARS 09882 | - | Pearl and Hermes Atoll, Hawai‘i (27°53.7636'N, 175°56.0316'W), 13 m depth, 03.VIII.2019, leg. H. Spalding & T. Williams (AM19) | MT039621 | MT039601 | MT039627 |
| ARS 09883 | - | Pearl and Hermes Atoll, Hawai‘i (27°57.468'N, 175°48.1248'W), 2 m depth, 04.VIII. 2019, leg. H. Spalding & T. Williams (AM26) | MT039622 | - | MT039628 |
| ARS 09884 | - | Pearl and Hermes Atoll, Hawai‘i (27°47.3952'N, 175°59.889'W), 12 m depth, 06.VIII.2019, leg. H. Spalding & B. Craig (AM60) | MT039623 | MT039602 | - |
| ARS 09885 | - | Pearl and Hermes Atoll, Hawai‘i (27°51.0924'N, 175°44.3214'W), 14 m depth, 09.VIII.2019, leg. H. Spalding & T. Williams (AM88) | MT039624 | MT039603 | - |
| ARS 09886 | - | Pearl and Hermes Atoll, Hawai‘i (27°57.9672'N, 175°46.467'W), 19 m depth, 09.VIII.2019, leg. H. Spalding & T. Williams (AM110) | - | MT039604 | MT039629 |
| ARS 09887 | - | Pearl and Hermes Atoll, Hawai‘i (27°54.6372'N, 175°54.2898'W), 17 m depth, 31.VII.2019, leg. H. Spalding & T. Williams (NWHI-803a) | - | MT039605 | - |
| ARS 09888 | - | Pearl and Hermes Atoll, Hawai‘i (27°54.6372'N, 175°54.2898'W), 17 m depth, 31.VII.2019, leg. H. Spalding & T. Williams (NWHI-804a) | MT039625 | MT039606 | MT039630 |
| ARS 09889 | BISH 776130 | Pearl and Hermes Atoll, Hawai‘i (27°57.468'N, 175°48.1248'W), 2 m depth, 31.VII.2019, leg. H. Spalding & T. Williams (NWHI-811-a) | - | MT039607 | - |
| ARS 10151 | BISH 776131 | Pearl and Hermes Atoll, Hawai‘i (27º47.5896'N, 175º59.8332'W), 14 m depth, 11.IX.2016, leg. L. Giuseffi (PHR-54) | MT039626 | - | - |
| ARS 10154 | BISH 776132, BISH 776133, HAW-43414 | Pearl and Hermes Atoll, Hawai‘i (27º47.3952'N, 175º59.889'W), 12 m depth, 01.VIII.2019, leg. T. Williams (NWHI-879) | - | - | - |
