# Supplementary material for: Taxonomic determination of the cryptogenic red alga, Chondria tumulosa sp. nov., (Rhodomelaceae, Rhodophyta) from Papahānaumokuākea Marine National Monument, Hawai‘i, USA: A new species displaying invasive characteristics
Source: PLoS One. 2020 Jul 7;15(7):e0234358. doi: 10.1371/journal.pone.0234358 (PMC7340295; doi:10.1371/journal.pone.0234358)
Supplement: S2 Table — (DOCX) [file pone.0234358.s002.docx]

**S2 Table.** **Accession data for sequences used in phylogenetic analyses.**

| Species | Voucher numbers | *rbcL* GenBank accession numbers | COI GenBank (or BOLD) accession numbers | SSU GenBank accession numbers |
| --- | --- | --- | --- | --- |
| *Acanthophora dendroides* | NCU-A-0000027 | MH388561 | - | - |
| *Acanthophora spicifera* | LS16 | - | - | MN626387.1 |
| *Acanthophora spicifera* | UA0152 | MH388556 | - | - |
| *Acrocystis nana* | SAP106293 | - | - | GU223764 |
| *Acrocystis nana* | UA0166 | MH388562 | - | - |
| *Alsidium corallinum* | PD4269 | MN165089 | - | - |
| *Alsidium seaforthii* | G001 | MH204653 | - | - |
| *Amansia dietrichiana* | UA0006 | MH388535 | - | - |
| *Amansia glomerata* | 03091 | - | - | HM582911.1 |
| *Amansia multifida* | GGS010 | MH388520 | - | - |
| *Amansia pinnatifida* | UA0013 | MH388536 | - | - |
| *Bostrychia intricata* | C3400.MX | KM502841 | - | - |
| *Bostrychia mixta* | Nightingale 2010 068 | MK185794 | - | - |
| *Bostrychia moritziana* | JW3660 | - | - | MF093912 |
| *Ceramium virgatum* | 2010-3-Csp4 | - | - | KP828754 |
| *Ceramium virgatum* | 288 | AF439289 | - | - |
| *Chondria arcuata* | ARS 00628 | MT039608 | HQ423044 | - |
| *Chondria armata* | SAP115359 | MG255051 | - | - |
| *Chondria atropurpurea* | FK012 | MH388516 | - | - |
| *Chondria baileyana* | FK001 | MH388512 | MH388698 | - |
| *Chondria baileyana* | GGS013 | MH388527 | - | - |
| *Chondria baileyana* | GWS011658 | KU564500 | - | - |
| *Chondria baileyana* | GWS032183 | - | KU564345 | - |
| *Chondria californica* | - | AY172578 | - | - |
| *Chondria californica* | ce123 | KF672856 | - | - |
| *Chondria californica* | LAF-07-15-00-1-1 | KF564781 | - | - |
| *Chondria capensis* | JFC1671 | MF094042 | - | - |
| *Chondria capensis* | D633 | KY927801 | - | - |
| *Chondria capensis* | D1004 | KY927799 | - | - |
| *Chondria capillaris* | SAP106295 | MG255052 | - | - |
| *Chondria* cf. *curdieana* | SAP115395 | MG843869 | - | - |
| *Chondria cnicophylla* | - | - | (ALSEU125-16) | - |
| *Chondria coerulescens* | 25428 | MF094043 | - | - |
| *Chondria coerulescens* | SANT-Algae-24340 | - | KF671147 | - |
| *Chondria collinsiana* | SP399.865 | GU330225 | - | - |
| *Chondria crassicaulis* | SAP115360 | MG255053 | - | - |
| *Chondria crassicaulis* | SAP115362 | - | - | MG272281 |
| *Chondria crassicaulis* | CQDHQW20111027-R9 | - | KC795910 | - |
| *Chondria dasyphylla* | - | CDU04021 | - | - |
| *Chondria dasyphylla* | FK007 | MH388513 | MH388700 | - |
| *Chondria decipiens* | ce121 | KF672855 | - | - |
| *Chondria decipiens* | UC2025838 | MG255056 | - | - |
| *Chondria expansa* | SAP115365 | MG255057 | - | - |
| *Chondria expansa* | SAP115366 | - | - | MG272239 |
| *Chondria intertexta* | SAP115364 | MG255059 | - | - |
| *Chondria littoralis* | ce102 | KF672853 | - | - |
| *Chondria littoralis* | ce106 | KF672854 | - | - |
| *Chondria mageshimensis* | SAP115367 | MG255060 | - | - |
| *Chondria polyrhiza* | LAF-5-12-06-2-15 | KF564787 | - | - |
| *Chondria ryukyuensis* | SAP115368 | MG255061 | - | - |
| *Chondria scintillans* | JML0048 | KF492775 | KF492717 | - |
| *Chondria scintillans* | RMAR2421 | - | KJ960536 | - |
| *Chondria scintillans* | RMAR3143 | - | KJ960537 | - |
| *Chondria* sp. | - | - | (GRHOD116-10) | - |
| *Chondria* sp. | - | - | (GRHOD332-10) | - |
| *Chondria* sp. | - | - | (GRHOD452-10) | - |
| *Chondria* sp*.* | - | MANC037-13 | - | - |
| *Chondria* sp. | PD0620 | - | - | MF093919 |
| *Chondria* sp. | PD0745 | - | - | MF093920 |
| *Chondria* sp. | PD1582 | - | - | MF093921 |
| *Chondria* sp. ARS-2011 | ARS 00629 | MT039613 | - | - |
| *Chondria* sp. ARS-2011 | ARS 01780 | - | HQ423002 | - |
| *Chondria* sp. ARS-2011 | ARS 01783 | - | HQ423059 | - |
| *Chondria* sp. ARS-2011 | ARS 02062 | MT039609 | - | - |
| *Chondria* sp. ARS-2011 | ARS 02063 | MT039610 | - | - |
| *Chondria* sp. ARS-2011 | ARS 02748 | MT039616 | - | - |
| *Chondria* sp. ARS-2011 | ARS 02916 | MT039618 | - | - |
| *Chondria* sp. ARS-2011 | ARS 03562 | MT039617 | HQ422964 | - |
| *Chondria* sp. ARS-2011 | ARS 03592 | MT039614 | - | - |
| *Chondria* sp. ARS-2011 | ARS 03588 | MT039611 | HQ422895 | - |
| *Chondria* sp. ARS-2011 | ARS 03726 | MT039615 | - | - |
| *Chondria* sp. ARS-2011 | ARS 03847 | MT039619 | - | - |
| *Chondria* sp. ARS-2011 | ARS 03857 | - | HQ423008 | - |
| *Chondria* sp. ARS-2011 | ARS 04516 | MT039612 | - | - |
| *Chondria* sp. ARS-2011 | ARS 04529 | MT039620 | - | - |
| *Chondria* sp. 1 JFC-2019 | HV06408 | MK125368 | - | - |
| *Chondria* sp. 1 SSut-2108 | SAP115363 | MG255062 | - | MG272240 |
| *Chondria* sp. | PD1759 | MF094046 | - | - |
| *Chondria* sp. SSut-2018a | SAP115391 | MG843867 | - | - |
| *Chondria* sp. | D479 | KY927806 | - | - |
| *Chondria* sp. WES-2014b | LAF-07-4-98-01-01 | KF564780 | - | - |
| *Chondria* sp. WES-2014 | ce86 | KF672851 | - | - |
| *Chondria tumulosa* sp. nov. | ARS 09882 | MT039601 | MT039621 | MT039627 |
| *Chondria tumulosa* sp. nov. | ARS 09883 | - | MT039622 | MT039628 |
| *Chondria tumulosa* sp. nov. | ARS 09884 | MT039602 | MT039623 | - |
| *Chondria tumulosa* sp. nov. | ARS 09885 | MT039603 | MT039624 | - |
| *Chondria tumulosa* sp. nov. | ARS 09886 | MT039604 | - | MT039629 |
| *Chondria tumulosa* sp. nov. | ARS 09887 | MT039605 | - | - |
| *Chondria tumulosa* sp. nov. | ARS 09888 | MT039606 | MT039625 | MT039630 |
| *Chondria tumulosa* sp. nov. | ARS 09889 | MT039607 | - | - |
| *Chondria tumulosa* sp. nov. | ARS 10151 | - | MT039626 | - |
| *Chondria succulenta* | NSW879470 | KY120336 | - | - |
| *Chondria tenuissima* | PD2129 | MF094050 | - | - |
| *Chondrophycus papilosus* | - | AF485807 | - | - |
| *Chondrophycus poiteaui* | From USA | EF061652 | - | - |
| *Cladurus elatus* | PD1176 | MF094051 | - | - |
| *Dasyclonium incisum* | JFC1784 | MF094056 | - | - |
| *Dasyclonium flaccidum* | UA0056 | MH388548 | - | - |
| *Dictyomenia harveyana* | GWS029521 | KU564482 | - | - |
| *Digenea arenahauriens* | UA0077 | MH388550 | - | - |
| *Digenea mexicana* | CNU080663 | MH514862 | - | - |
| *Digenea rafaelii* | CNU037775 | MH514867 | - | - |
| *Digenea simplex* | PD1820 | - | - | MF093926 |
| *Digenea simplex* | Dig sim | - | - | HM560626 |
| *Digenea simplex* | RM0001 | - | MG030761 | - |
| *Dipterosiphonia australica* | PD1107 | - | - | MF093928.1 |
| *Dipterosiphonia dendritica* | PD1654 | MF094058 | - | - |
| *Dipterosiphonia rigens* | CI003 | MH388509 | - | - |
| *Epiglossum smithiae* | UA0030 | MH388544 | - | - |
| *Herposiphonia insidiosa* | CUK10410 | KT825868 | - | - |
| *Herposiphonia tenella* | 25835 | MF094078 | - | - |
| *Herposiphonia versicolor* | PD0852 | - | - | MF093930.1 |
| *Heterocladia caudata* | - | - | - | AF203891.1 |
| *Janczewskia morimotoi* | OK228 | JX828128 | - | - |
| *Laurencia complanata* | - | AF465813 | - | - |
| *Laurencia filiformes* | NOW0416 89r | - | - | MF579979 |
| *Laurencia flexilis* | - | AF489860 | - | - |
| *Laurencia flexuosa* | - | AF465915 | - | - |
| *Laurencia intricata* | - | AF465809 | - | - |
| *Laurencia obtusa* | - | AF281881 | - | - |
| *Laurencia snackeyi* | JC0032 | - | - | MF093932 |
| *Laurencia* sp. | SWD1 | - | - | MN447109 |
| *Lembergia allanii* | - | - | - | AF373215.1 |
| *Leptosiphonia schousboei* | CH826 | JX828133 | - | - |
| *Lophocladia trichoclados* | - | - | - | GQ504015.1 |
| *Lophocladia trichoclados* | 662102 | MH388508 | - | - |
| *Lophosiphonia obscura* | CUK11188 | KT825865 | - | - |
| *Lophosiphonia obscura* | 26325 | - | - | MF093963 |
| *Lophosiphonia simplicissima* | 24157 | MF094088 | - | - |
| *Melanothamnus collabens* | CH2526 | JX828157 | - | JX828188.1 |
| *Melanothamnus harveyi* | PD0890 | - | - | MF093937.1 |
| *Melanothamnus* sp. | OShj0705-18 | MH200860 | - | - |
| *Micropeuce strobiliferum* | - | - | - | AF203896 |
| *Nanopera merrifieldiae* | PD1789 | MF094092 | - | - |
| *Neochondria ammophila* | SAP115347 | - | - | MG272241 |
| *Neochondria ammophila* | SAP115369 | - | - | MG272242 |
| *Neochondria ammophila* | SAP115370 | MG255065 | - | MG272243 |
| *Neochondria ammophila* | SAP115371 | - | - | MG272244 |
| *Neochondria nidifica* | UC2026095 | MG255067 | - | MG272245 |
| *Neorhodomela larix* | AC136 | GQ252553 | - | - |
| *Odonthalia floccosa* | - | - |  | AY617141.1 |
| *Odonthalia washingtoniensis* | AC107 | GQ252561 |  |  |
| *Ophidocladus simpliciusculus* | CH827 | - |  | JX828177.1 |
| *Ophidocladus simpliciusculus* | SANTA25413 | MG975684 |  | - |
| *Osmundaria fimbriata* | JW2841 | - | MF093991 | - |
| *Osmundaria obtusiloba* | 03151 | - | - | HM582914.1 |
| *Osmundaria obtusiloba* | UA0089 | MH388551 | - | - |
| *Osmundaria prolifera* | UA0024 | MH388541 | - | - |
| *Osmundaria spiralis* | UA0025 | MH388542 | - | - |
| *Osmundea hybrida* | - | - | - | GU223794.1 |
| *Osmundea pinnatifida* | 1039 | AF281876 | - | - |
| *Osmundea splendens* | - | AY172576 | - | - |
| *Palisada flagellifera* | TFC Phyc N#13127 | - | - | - |
| *Pleurostichidium falkenbergii* | - | - | - | AF251511.1 |
| *Pleurostichidium falkenbergii* | - | MH853471 | - | - |
| *Pollexfenia lobata* | GWS015810 | KU564506 | - | - |
| *Polyostea robusta* | GWS003369 | KU876561 | - | - |
| *Polyshiponia stricta* | CH052 | - | - | AF427535 |
| *Polyzonia elegans* | JFC1562 | MF094099 | - | MF093953.1 |
| Pterosiphonia dendroidea | CUK15436 | KU252579 | - | - |
| *Pterosiphonia pennata* | CH977 | - | - | JX828187.1 |
| *Rhodomela confervoides* | - | - | - | AY617145.1 |
| *Rhodomela virgata* | GWS009336 | KU564489 | - | - |
| *Sonderella linearis* | PD1151 | NC_035289 | - | MF093955.1 |
| *Thaumatella adunca* | GWS015067 | MF120887 | - | - |
| *Thaumatella adunca* | PD1388 | - | - | MF093958.1 |
| *Tolypocladia glomerulata* | PD1825 | MF101467 | - | MF101467 |
| *Ululania stellata* | ARS03566 | - | - | GU223745.1 |
| *Vertebrata lanosa* | - | - | - | AY617143.1 |
| *Vertebrata reptabunda* | SANTAlgae25139 | - | - | KX499574.1 |
| *Wilsonosiphonia howei* | CUK10056 | KT825864 | - | - |
| *Womersleyella pacifica* | CUK10968 | KT825866 | - | - |
| *Womersleyella setacea* | - | - | - | AF427537.1 |
| *Wrightiella tumanowiczii* | Wri_tuman | EU492922 | - | - |
| ***OUTGROUPS*** |  |  | - |  |
| *Acrosorium ciliolatum* | HV06329 | - | - | MK125455 |
| *Acrosorium ciliolatum* | PC280917020 | MH349419 | - | - |
| *Caloglossa beccarii* | JW4523 | - | - | MF093916 |
| *Caloglossa intermedia* | JW3535 | - | - | MF093917 |
| *Caloglossa intermedia* | K42 | JN845514 | - | - |
| *Caloglossa monosticha* | JW3046 | - | - | MF093918 |
| *Caloglossa monosticha* | K130 | HM775469 | - | - |
| *Dasya baillouviana* | GH02 | FM993090 | - | - |
| *Dasya naccarioides* | PD888 | - | - | MF093923 |
| *Dipterocladia arabiensis* | - | NC_035257 | - | MF093927 |
| *Platysiphonia delicata* | - | NC_035258 | - | MF093942 |
| *Spyridia clavata* | SP470212 | - | - | MH883046 |
| *Spyridia hypnoides* | TC2836 | KU756101 | - | - |
| *Taenioma perpusillum* | PD1676 | NC_035295 | - | MF093957 |
| *Thuretia quercifolia* | PD1024 | NC_035286 | - | MF093959 |
